# NHSE Regional International Medical Graduates Conference for Yorkshire and Humber School of Psychiatry

**DOI:** 10.1192/bjo.2025.10269

**Published:** 2025-06-20

**Authors:** Christiana Elisha-Aboh, Anilkumar Pillai, Nazish Hashmi, Senaka Weerakoon, Theresa Ugalahi

**Affiliations:** 1 Tees, Esk and Wear Valley Foundation Trust, York, United Kingdom; 2 Bradford District Care Trust, Bradford, United Kingdom; 3 Leeds and York Partnership Foundation Trust, Leeds, United Kingdom; 4 Humber Teaching NHS Foundation Trust, Hull, United Kingdom

## Abstract

**Aims:** International Medical Graduates (IMGs) represent over 30% of the NHS workforce. They play a vital role in addressing healthcare shortages and enhancing cultural diversity. They are a significantly mixed group in terms of their experience, skills and overall development. Due to differing systems, IMGs often require additional support. The right support ensures that they achieve their professional goals, highlighting the need for targeted conferences.

The conference aimed to facilitate collaboration and knowledge exchange between trainers and trainees, fostering mutual understanding. It also supported IMGs by providing insights into navigating the UK healthcare system while addressing their challenges through data discussion.

**Methods:** The “Untying the Knots” conference, held from 09:00 to 16:30, registered approximately 100 doctors and featured a poster competition. It included 21 speakers covering diverse topics, focusing on Language, Culture and the Clinical Encounter. International speakers shared insights on training in India and Nigeria, while sessions addressed IMG challenges and personal journeys. Pre- and post-workshop surveys evaluated effectiveness and informed future planning.

**Results:** 89% of those who attended were IMGs, with only 7% being trainers. Majority of the attendees were core trainees or Trust staff grades (28% each). There were participants from all 7 Trusts in the region and one Trust outside the region. Most achieved their primary degree in India, followed by Pakistan, then Nigeria. Most seemed to have a good confidence level (Predominantly or very confident) on topics relating to differential attainment, language/culture, portfolio management and compassionate leadership and less confident (slightly or fairly confident) about issues relating to the impact of patient safety incidents, navigating GMC referrals/SUIs and CESR pathway. The feedback was that people enjoyed the conference and 90% would attend again with 66% wanting it as a yearly event. 96% felt the conference was well organised with the presentations and networking being the most attractive components. 86% would prefer a face-to-face conference in the future and 10% a hybrid event.

**Conclusion:** Overall, the feedback was highly positive, with most attendees expressing interest in future events and suggesting it be held annually. The organisation, presentations, and networking opportunities were particularly well-received, with a strong preference for face-to-face events moving forward. These findings will help shape future conferences to better meet the needs of international medical graduates.

Upcoming conferences will implement feedback, explore topics of interest further like supporting the CESR pathway, and facilitating discussions on GMC referrals and Serious Untoward Incidents (SUIs).

